# Comparing clinical outcomes of piperacillin-tazobactam administration and dosage strategies in critically ill adult patients: a systematic review and meta-analysis

**DOI:** 10.1186/s12879-020-05149-6

**Published:** 2020-06-20

**Authors:** Sarah Fawaz, Stephen Barton, Shereen Nabhani-Gebara

**Affiliations:** Faculty of Science, Engineering and computing, Kingston University, Penrhyn Rd, London, Kingston upon Thames KT1 2EE UK

**Keywords:** Critically ill patients, Continuous infusions, Prolonged infusion, Intermittent infusion and clinical efficacy

## Abstract

**Background:**

Recently, continuous administration of piperacillin-tazobactam has been proposed as a valuable alternative to traditional intermittent administration especially in critically ill patients. However, antibiotic dosing remains a challenge for clinicians as antibiotic dosing regimens are usually determined in non-critically ill hospitalized adult patients. The aim was to conduct a systematic review to identify and highlight studies comparing clinical outcomes of piperacillin tazobactam dosing regimens, continuous/prolonged infusion vs intermittent infusion in critically ill patients. Meta-analyses were performed to assess the overall effect of dosing regimen on clinical efficacy.

**Methods:**

Studies were identified systematically through searches of PubMed and Science Direct, in compliance with PRISMA guidelines. Following the systematic literature review, meta-analyses were performed using Review Manager.

**Results:**

Twenty-three studies were included in the analysis involving 3828 critically ill adult participants in total (continuous/prolonged infusion = 2197 and intermittent infusion = 1631) from geographically diverse regions. Continuous/prolonged resulted in significantly: higher clinical cure rates (Odds Ratio 1.56, 95% Confidence Interval 1.28–1.90, *P* = 0 .0001), lower mortality rates (Odds Ratio 0.68, 95% Confidence Interval 0.55–0.84, *P* = 0 .0003), higher microbiological success rates (Odds Ratio 1.52, 95% Confidence Interval 1.10–2.11, *P* = 0.01) and decreasing the length of hospital stay (Mean Difference − 1.27, 95% Confidence Interval − 2.45—0.08, *P* = 0.04) in critically ill patients.

**Conclusion:**

Results from this study show that there is a significant level of evidence that clinical outcome in critically ill patients is improved in patients receiving piperacillin-tazobactam via continuous/prolonged infusion. However, more rigorous scientific studies in critically ill patients are warranted to reach a sufficient level of evidence and promote further implementation of C/PI as a dosing strategy.

## Background

Recently, continuous administration of piperacillin-tazobactam has been proposed as a valuable alternative to traditional intermittent administration especially in critically ill patients. However, correct antibiotic dosing remains a challenge for clinicians as antibiotic dosing regimens are usually determined in non-critically ill hospitalized adult patients. Patient that are in intensive care units (ICU) differ from other hospitalized patients in terms of pathophysiology and disease severity; these factors not only affect metabolism but also drug pharmacokinetics/pharmacodynamics (PK/PD) behaviour. Critically ill patients also have an increased risk (5–10 times more likely) of having or developing infections and infectious complications than those in general wards [1].

Dosing strategies that have been validated in patient populations that are non-critically ill fail to consider the substantial changes in organ function that occur with critical illness [2]. Augmented renal clearance of antibiotics is increasingly reported in critically ill patients. Antibiotic dosing concentrations will vary greatly within intensive care patients with normal kidney function or renal failure as the pharmacokinetic target attainment is dependent on kidney function [3]. Given the enhanced renal elimination reported in critically ill patients, antimicrobial dosing requires extensive consideration due to important clinical consequences as accurate and timely drug exposure is essential for clinical success. The augmented renal clearance is possibly associated with the (1) immune response to infection, (2) inflammation to fluid loading and, (3) use of vasoactive medications. An increase in both cardiac output and blood flow is therefore observed, leading to enhanced glomerular filtration that results in sub-therapeutic piperacillin-tazobactam concentrations due to substantial drug elimination [4].

The optimisation of antimicrobial agents is a relatively unexplored area where further research is needed. Continuous infusions (CI) and prolonged infusions (PI) of piperacillin-tazobactam has been directly linked to improved clinical outcome displaying capabilities such as lowering the possibility of resistance and decreasing mortality [2, 5, 6]. The aim here is to systematically review the literature comparing the clinical outcome of piperacillin tazobactam dosing regimens, continuous/prolonged infusion C/PI and II.

## Methods

### Literature search

A systematic review of the literature was conducted [7–10]; references published between 1998 and 2019 were acknowledged through searches on PubMed and Science Direct, in compliance with PRISMA guidelines. Search terms used were: (penicillin OR penicillins OR piperacillin OR tazobactam OR piperacillin-tazobactam OR piperacillin/tazobactam) AND (intermittent OR bolus OR short OR prolonged OR extended OR continuous) AND (infusion OR duration OR administration OR interval OR dosing) AND (intensive care OR ICU OR critically ill OR critical care OR septic shock OR sepsis OR severe sepsis).

However, like any database, their coverage is not complete, therefore the authors retrieved additional articles using supplementary approaches such as manual searching of journals, Google Scholar and checking reference lists of articles to identify additional text. A full review of published studies was implemented addressing and comparing clinical outcome of IV piperacillin-tazobactam dosing regimens administered to infected critically ill patients. The last search was on the 1st of August 2019 [PROSPERO registration number: CRD42019117303].

### Study selection

Initially, all articles reporting comparative outcomes of critically ill patients treated with C/PI versus II piperacillin-tazobactam were considered eligible. The eligibility criteria were separated into two components: study characteristics and report characteristics. Study eligibility criteria included the types of a) studies, b) participants, c) interventions and d) outcome measures; these measures are presented in Table 1. Report eligibility criteria included: publications written in English language, study status is “published” and inclusion of both old and new data. Exclusion criteria included: Pharmacoeconomic studies, non-human subjects, non-adult subjects, non-critically ill subjects, non-English language studies and pilot studies. Systematic reviews, meta-analysis and editorials were also excluded.

**Table 1:** Showing eligibility criteria for study selection process

Eligibility Criteria	
a) Studies	Prospective and retrospective trials/studies comparing/evaluating clinical efficacy or clinical outcome of piperacillin/tazobactam administered via CI vs II in critically ill patients. Pilot studies excluded
b) Participants	Critically ill adult participants aged 18 and over suffering from documented bacterial infection and requiring treatment with piperacillin-tazobactam. Non-adult, non-human and non-critically ill patient studies were excluded.
c) Interventions	Studies comparing the beneficial and harmful/limiting effects of CI and II. Infusions of all types (CI, PI and II), dose and regimen are adequate for the review. Pharmacoeconomic studies were also excluded.
d) Outcome measures	All studies were eligible if specifically related to clinical outcome/efficacy of dosing regimens. All outcomes were included to reduce risk of bias as a consequence of selective reporting.

*CI* Continuous infusion, *II* Intermittent infusion

### Data analysis

A data extraction form was developed based on Cochrane data extraction template. The information extracted from each of the included studies consisted of:

1. Characteristics of participants (didn’t necessarily comprise characteristics such as age and sex however, includes characteristics such as the disease patient is diagnosed with and the method of diagnosis) and the eligibility criteria (inclusion and exclusion measures);
2. The type of intervention – mode of administration, continuous vs intermittent dosing (including the drug, dose, duration of infusion and frequency);
3. Type of outcome measure (including clinical outcome and clinical efficacy in terms of clinical cure).

One reviewer extracted the following data from included studies (S.F); the second and third reviewers verified the relevance of the extracted information (S.N-G and S.B). Variances in opinions were resolved by discussion between the three reviewers.

### Risk of Bias and study quality assessment

Methodological assessment of included RCTs was undertaken using the Cochrane risk of bias tool. Two reviewers individually assessed the risk of bias (S.F and S.N-G) with disagreements resolved by a third reviewer (S.B). Six domains of bias were assessed including: (1) random sequence generation, (2) allocation concealment, (3) blinding of participants and personnel, (4) incomplete outcome data, (5) selective reporting and (6) other biases. Publication bias was evaluated using funnel plots.

The methodological quality of included RCT’s was assessed with the Jadad Scale [11] that evaluated the trial’s randomisation, double blinding and reports of withdrawals and dropouts. An overall score of 0–5 points was assigned, where an overall score of three and above was regarded as adequate trial quality.

The Newcastle-Ottawa Scale is a quality assessment tool for selection, comparability and outcome assessment used to assess the quality of included observational studies (retrospective and prospective) [12]. Studies scoring more than six stars are considered as being good quality.

No studies were excluded on the basis of quality assessment however their quality scores were taken into account when describing results.

### Statistical analysis

Meta-analysis was performed using Review Manager for Windows Version 5.3 to compare the clinical efficacy of C/PI vs II in terms of clinical cure, mortality, microbiological cure rates, adverse events and length of hospital stay. Pooled odds ratio (OR) and 95% confidence intervals (C.I) were calculated for dichotomous data, taking into account all outcomes from included studies. Pooled mean difference (MD) and 95% C.I were calculated for continuous data. Statistical heterogeneity was assessed by employing *χ*^2^ test and I^2^ statistic. The presence of heterogeneity between studies was assessed by *χ*^2^ test (P < 0.10 indicates significant heterogeneity) and the extent of the inconsistencies was considered using I^2^ statistic (I^2^ > 70% indicates considerable heterogeneity). The pooled outcomes were calculated using Mantel-Haenszel fixed effect model when there was no significant heterogeneity otherwise the random effects model was chosen. ‘Emergence of resistance’ was narratively reviewed instead of statistical analysis considering the few sample sizes included.

## Results

### Search results

The search of PubMed and Science Direct provided 199 citations. Of these, 154 studies were excluded following review of the abstracts, as they did not meet the inclusion criteria. Twenty articles were discarded after reviewing the full article due to the following reasons: non-human (*n* = 2), on non-critically ill (*n* = 10) and children (*n* = 8) subjects. A further four studies were eliminated due to the focus being on pharmacoeconomics and renal replacement therapy.

**Fig. 1:**
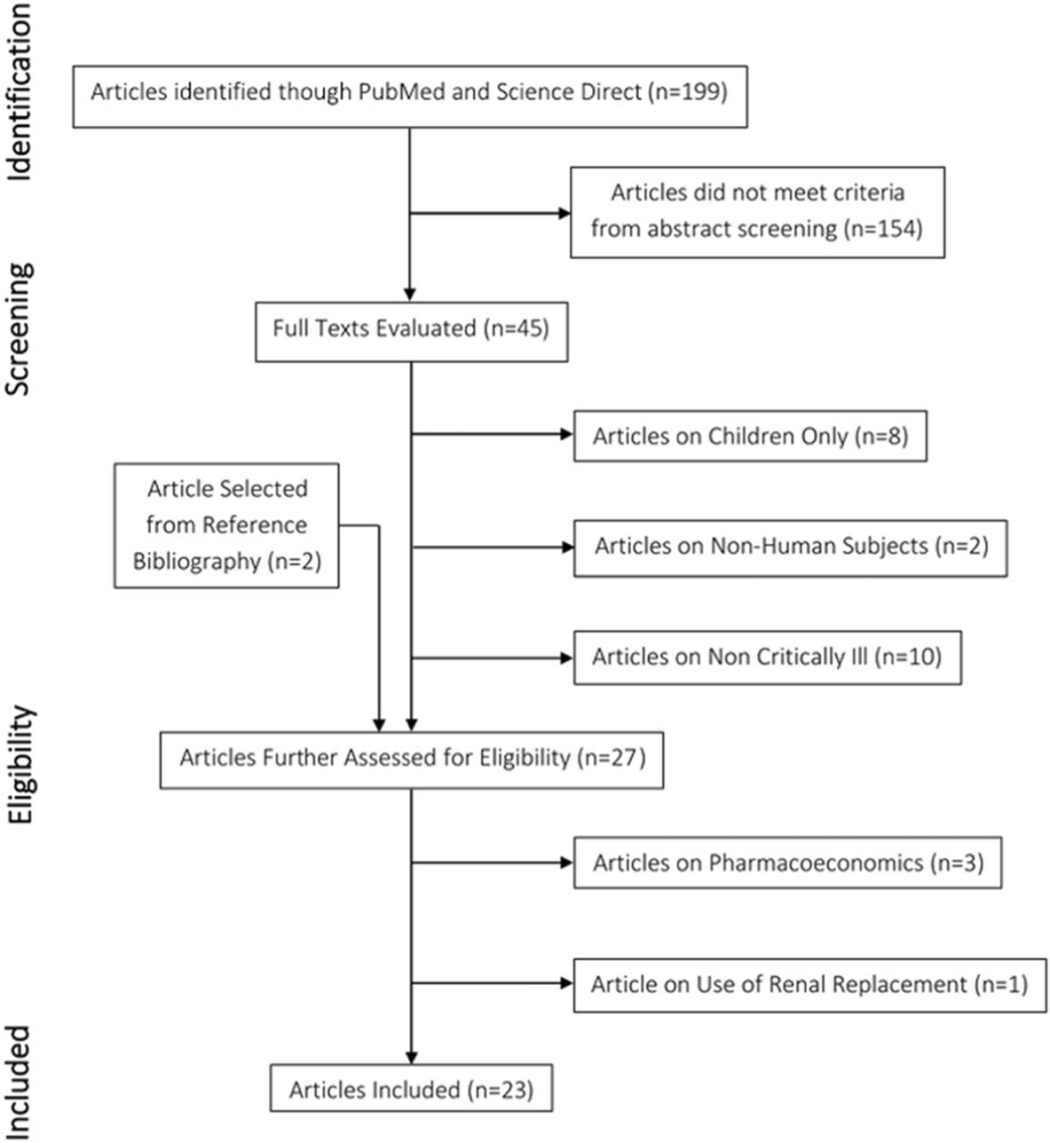
Flow diagram illustrating the selection process for included studies

An additional two studies that met the inclusion criteria were acknowledged through checking references of relevant studies. Twenty-three studies met the described inclusion criteria and were included in the systematic review [13–34]. The article selection process is illustrated in Fig. 1 and selected studies comparing clinical outcome between CI and II of piperacillin are listed in Table 2. Characteristics of included studies comprising of demographic characteristics, C/PI and II dosage, drug regimen treatment results as well as study outcomes and suggestions were extracted from all studies and summarised (Table 2). Out of the twenty-three studies included, only an abstract (and no full article) could be obtained for four of the studies [19, 20, 25, 26].

**Table 2:** Characteristics of studies comparing outcomes for continuous versus intermittent infusions of piperacillin-tazobactam

Study	Study Design/Patient Population	Age (avg)	Gender	Dosage	Clinical Cure n/N (%)	Mortality n/N (%)	Outcome/Suggestions
Grants et al. 2002 [13] *USA*	Prospective cohort study 98 ICU patients	CI - 66 II – 65	F - 37 M - 61	CI (*n* = 47) – 2.25 g LD + 9 g DD over 24 h CI II (*n* = 51) – 3.375 g every 6 h over 30 min II	CI- 44/47 (94%) II- 42/51 (82%)	CI- 1/47 (2.1%) II- 5/51 (9.8%)	CI provided equivalent clinical and microbiologic to II. CI is a cost-effective alternative to II. CI is well tolerated resulting in CC.
Lau et al. 2006 [14] *USA*	Randomised control trial 167 patients with gram +/− bacteria	CI – NR II – NR	F - NR M - NR	CI (*n* = 81) – 13.5 g over 24 h CI II (*n* = 86) - 3.375 g every 6 h over 30 min II	CI- 70/81 (86%) II- 76/86 (88%)	CI- 1/130 (0.8%) II- 3/132 (2.3%)	CI are a same and reasonable alternate mode of administration. No differences in bacteriological response by pathogen was noted between CI and II.
Rafati et al. 2006 [15] *Iran*	Randomised control trial 40 Septic, critically ill patients	CI - 50.1 II – 48	F - 13 M - 27	CI (*n* = 20) – 2.25 g LD + 9 g DD over 24 h CI II (*n* = 20) – 3.375 g every 6 h over 30 min II	CI- 15/20 (75%) II- 16/20 (70%)	CI- 5/20 (25%) II- 6/20 (30%)	Clinical efficacy as a CI is superior to that with II. CI significantly reduces severity of illness resulting in clinical cure.
Lodise et al. 2007 [16] *USA*	Retrospective cohort Study 194 ICU patients with Pa	PI - 63.2 II – 63.2	F - 75 M - 119	PI (*n* = 102) – 3.375 g every 8 h over 4 h PI II (*n* = 92) - 3.375 g every 4-6 h over 30 min II	PI- NR II- NR	PI- 5/41 (12.2%) II-12/92 (13%)	No difference in baseline clinical characteristics were noted between the two dosing regimens, however, mortality rates were significantly lower with PI.
Roberts et al. 2009 [17] *Austrailia*	(*) Randomised control trial 16 Critically ill adult patients	CI - 30 II – 41	F - 5 M - 11	CI (*n* = 8) – 4.5 g LD + 9 g DD over 24 h CI II (*n* = 8) – 4 g every 6-8 h over 20 min II	CI- 8/8 (100%) II- 8/8 (100%)	CI- 0/8 (0%) II- 0/8 (0%)	Administration by CI with initial loading dose achieves superior PD target and CC when compared with conventional II
Lorente et al. 2009 [18] *Spain*	(*) Retrospective cohort study 83 ICU patients suffering VAP	CI - 63.2 II – 61.8	F - 18 M - 65	CI (*n* = 37) – 4.5 g LD + 18 g DD over 24 h CI II (*n* = 46) – 4 g every 6 h over 30 min II	CI- 33/37 (89.2%) II- 26/46 (56.2%)	CI- 8/37 (21%) II- 14/46 (30.4%)	Higher clinical efficacy achieved by continuous infusion. Higher DD reached target concentration for pathogens with higher MIC’s
Li et al. 2010 [19] *China*	Randomised control trial 66 patients with severe pneumonia	PI – NR II – NR	F - NR M - NR	CI (*n* = 28) - 4.5 g every 8 h over 8 h CI II (*n* = 31)- 4.5 g every 8 h over 30 min II	CI- 24/32 (75%) II- 17/34 (50%)	CI- NR II- NR	Results obtained from the study suggest clinical advantages of CI compared with II administration in patients suffering with severe pneumonia.
Rose et al. 2011 [35] *USA*	Retrospective cohort study 90 ICU patients	PI - 58.4 II – 60.4	F - 13 M - 77	PI (*n* = 54) – 3.375 g every 8–12 h over 4 h PI II (*n* = 36) - 3.375 g every 8–12 h over 30 min II	PI- NR II- NR	CI- NR II- NR	PI reduced: (1) days of therapy in ICU, (2) time spent on ventilator, (3) length of ICU and hospital stay and, (4) mortality.
Ye et al. 2011 [20] *China*	Randomised control trial 66 ICU patients, gram (−) bacteria	PI – NR II – NR	F - NR M - NR	PI (*n* = 35) - 4.5 g every 8 h over a 3 h PI II (*n* = 31) – 4.5 g every 8 h over 30 min II	PI- 24/35 (68.6%) II- 13/31 (41.9%)	PI- 8/35 (22.9%) II- 8/31 (25.8%)	Prolonged infusion is superior to traditional regimens and should be recommended as empirical therapy for gram (−) bacteria
Yost et al. 2011 [21] *USA*	Retrospective cohort study 270 ICU patients with Pa	PI - 65 II – 62	F - 19 M - 141	PI (*n* = 186) - 3.375 g every 8 h over 4 h PI II (*n* = 84) - dose not recorded, 30 min II	PI- 171/186 (90.3%) II- 67/84 (79.8%)	PI- 18/186 (9.7%) II- 17/84 (20.2%)	Pharmacodynamic dosing via PI’s of piperacillin-tazobactam demonstrated positive outcome compared with II. PRT need to further verify findings.
Fahmi et al. 2012 [22]	Quasi experimental study 61 ICU patients with VAP	PI – NR II – NR	F - NR M - NR	PI (*n* = 31) – 3.375 g every 8 h over a 4 h PI II (*n* = 30) - 3.375 g every 6 h over 30 min II	PI- NR II- NR	PI- NR II- NR	No significant difference in clinical outcome of PI and II. Suggest administration by PI or II according to MIC of organism.
Pereira et al. 2012 [23] *Portugal*	Retrospective cohort study 346 ICU patients	CI – NR II – NR	F - NR M - NR	CI (*n* = 173) – Majority 18 g DD, every 8 h II (*n* = 173) – Majority 18 g DD, 30 min II	CI- 124/173 (71.7%) II- 124/173 (71.7%)	CI- 49/173 (28.3%) II- 49/173 (28.3%)	Clinical efficacy of piperacillin-tazobactam dosing was independent of the mode of administration. CI is not associated with a decrease in mortality.
Lee et al. 2012 [24] *USA*	Retrospective cohort study 148 ICU patients	PI – 64 II – 69.6	F - 64 M - 84	PI (*n* = 68) – 3.375 g every 8 h over 4 h PI II (*n* = 80)- 2.25 g every 6 h over 30 min II	PI- 55/68 (81%) II- 50/80 (62%)	PI- 13/68 (19.1%) II- 30/80 (37.5%)	Results suggest improved 30-day mortality in ICU patients treated via PI vs CI. Clinical benefits of PI at lower MIC’s are less substantial compared with more RO.
Waxier et al. 2012 [25] *-*	Retrospective cohort study 400 ICU patients	PI – NR II – NR	F - NR M - NR	PI (*n* = 200) - dose not recorded, over 4 h PI II (*n* = 200) - dose not recorded, over 30 min II	PI- NR II- NR	PI- NR II- NR	PI patients received fewer doses and demonstrated decreased morbidity and mortality; results however are not SS so larger prospective studies are needed.
Lu et al. 2013 [26] *China*	Randomized control trial 50 patients with HAP	PI – NR II – NR	F - NR M - NR	PI (*n* = 25) - 4.5 g every 6 h over a 3 h PI II (*n* = 25) - 4.5 g every 6 h over 30 min II	PI- 22/25 (88%) II- 20/25 (80%)	PI- NR II- NR	PI’s of piperacillin-tazobactam for gram negative bacteria with high MIC values, like HAP, provide stable plasma concentration and curative clinical effect.
Cutro et al. 2014 [27] *USA*	Retrospective cohort study 843 patients suffering from sepsis	PI – NR II – NR	F - NR M - NR	PI (*n* = 662) – 2.25-3.375 g every 6-12 h over 4 h PI II (*n* = 181) – 2.25-4.5 g every 8-12 h over 30 min II	PI- 540/662 (81.6%) II- 145/181 (80.1%)	PI- 72/662 (10.9%) II- 25/181 (13.8%)	No significant difference between the two dosing regimens was observed in terms of mortality or clinical cure however PI resulted in shorter duration of therapy.
Jamal et al. 2015 [28] *Malaysia*	(*) Randomised control trial 16 ICU patients	CI - 44 II – 62.5	F - 4 M - 12	CI (*n* = 8) - 2.25 g LD + 9 g DD over 24 h CI II (*n* = 8) – 2.25 g every 6 h over 30 min II	CI- 6/8 (75%) II- 6/8 (75%)	CI- 0/8 (0%) II- 0/8 (0%)	CI is advantageous in the presence of more resistant pathogens as it allows achievement of rapid and consistent piperacillin-tazobactam concentrations.
Abdul et al. 2016 [33] *Malaysia*	(*) Randomised control trial 85 ICU patients	CI - 54 II – 56	F - 27 M - 58	CI (*n* = 38) – dose not recorded II (*n* = 47) – dose not recorded	CI- 22/38 (58%) II- 15/47 (32%)	CI- 7/38 (18.4%) II- 20/47 (42.6)	Results showed that CI piperacillin-tazobactam demonstrated higher clinical cure rates and better PK/PD target attainment compared to II.
Schmees et al. 2016 [31] *USA*	Retrospective cohort study 113 ICU patients	PI - 68 II – 59.4	F - 47 M - 66	PI (*n* = 61) – 3.375-4.5 g every 8-12 h II (*n* = 52) – dose not recorded	PI-31/61 (50.8%) II-22/52 (42.3%)	PI-9/61 (14.8%) II-11/52 (21.1%)	Mortality rates and length of hospital stay were significantly lower in PI patients. PI improves patient outcomes while maintaining patient safety.
Cortina et al. 2016 [29] Spain	Randomised control trial 78 Patients with suspected Pa	CI - 64.3 II – 63.8	F - 32 M - 46	CI (*n* = 40) – 2.25 g LD + 8 g DD over 24 h CI II (*n* = 38) – 4.5 g every 8 h over 30 min II	CI- 20/40 (50%) II- 18/38 (47.4%)	CI- 0/40 (0%) II- 1/38 (2.6%)	No SS difference in efficacy between CI & II. Data indicates better performance of II than CI. II cure rates almost doubled CI.
Winstead et al. 2016 [30] *USA*	Retrospective cohort study 181 patients, gram (−) bacteria	PI - 65.1 II – 68.2	F - 99 M - 82	PI (*n* = 86) – 4.5 g LD + 3.375 g every 6 h over 3 h PI II (*n* = 95) - 4.5 g every 8 h over 30 min II	PI- NR II- NR	PI- 7/86 (8.1%) II- 6/95 (6.3%)	No SS difference in the primary outcome of mortality and length of hospital stay, however, 30-day hospital re-admission was significantly reduced in PI patients.
Bao et al 2017 [32] *China*	Randomised control trial 50 patients with HAP	PI - 69.75 II – 67.04	F - 21 M - 29	PI (*n* = 25) – 4.5 g every 6 h over a 3 h PI II (*n* = 25) – 4.5 g every 6 h over 30 min II	PI- 22/25 (88%) II- 20/25 (80%)	PI- 0/25 (0%) II- 0/25 (0%)	Dosing regimen had no impact on adequacy of treatment and that PI is as effective as II. PI is potentially a more cost-effective alternative to II.
Fan et al 2017 [34] *China*	Prospective cohort study 367 ICU patients	PI - 69 II – 70	F - 120 M - 247	PI (*n* = 182) - 4.5 g every 8-12 h over 4 h PI II (*n* = 185) - 4.5 g every 8-12 h over 30 min II	PI- NR II- NR	ssssssssPI- 21/182 (11.5%) II- 29/185 (15.6%)	No significant difference between the two dosing regimens in terms of mortality rate and length of hospital stay

*ICU* Intensive care unit, *CI* Continuous infusion, *II* Intermittent infusion, *PI* Prolonged infusion, *F* Female, *M* Male, *MIC* Minimal inhibition concentration, *LD* Loading dose, *DD* Daily dose, *VAP* Ventilator-associated pneumonia, *PD* Pharmacodynamic, *CC* Clinical cure, *Pa* P*seudomonas aeruginosa*, *SS* Statistically significant, *PRT* Prospective randomised trials, *RO* Resistant organisms, *HAP* Hospital acquired pneumonia, *NR* Not recorded; (*) = studies that reported SOFA score

### Definitions

‘Clinical cure’ was defined as ‘the complete resolution of clinical signs and symptoms of infection, with no new signs or symptoms associated with the original infection’ [32, 36].

‘Microbiological cure’ was defined as ‘the eradication and presumed eradication of organisms at the infection site’ [36].

‘Adverse events’ were defined as ‘any unexpected medical occurrences in patients administered piperacillin-tazobactam caused by either the drug or dosing regimen being received’ [36].

### Study characteristics

The type of studies included in the systematic review and meta-analysis were RCT’s (*n* = 10), observational cohort studies (*n* = 12; retrospective *n* = 10, prospective *n* = 2) and a Quasi-experimental study (non-randomised trial) (*n* = 1).

### Study quality

The quality of the majority of RCT’s included was moderate to high (Table 3). According to the Jadad scale, seven out of ten RCT’s (70%) obtained a score of three and above. The studies by Ye [20] and Lu [26] had a score of one and two respectively due to retrieval of only the abstract (full text unavailable). Rafati [15] received a score of two as the article did not describe randomisation method and study was not blinded. All observational studies assessed using the Newcastle Ottawa Scale scored eight or nine stars and recognised as being of high quality (Table 4).

**Table 3:** Quality assessment of randomised control trials in meta-analysis based on the Jadad Scale

Quality assessment of RCT’s	Lau [14]	Rafati [15]	Robert [17]	Li [19]	Ye [20]	Lu [26]	Jamal [28]	Abdul [33]	Cotrina [29]	Bao [32]
	**2006**	**2006**	**2009**	**2010**	**2011**	**2013**	**2015**	**2016**	**2016**	**2017**
^(1)^ Described as randomised	1	1	1	1	1	1	1	1	1	1
^(2)^ Described as double blind	0	0	0	0	0	0	0	0	1	0
^(3)^ Description of withdrawals	1	1	1	1	0	1	1	1	1	1
^(4)^ Randomisation method described	1	0	1	1	0	0	1	1	1	1
^(5)^ Double blinding method described	0	0	0	0	0	0	0	0	1	0
Score (−/5)	**3/5**	**2/5**	**3/5**	**3/5**	**1/5**	**2/5**	**3/5**	**3/5**	**5/5**	**3/5**

*RCT’s* Randomised Control Trials

Randomisation:

*Up to two points are given*^(1)^*: described as randomised (yes = 1) (no = 0) and*^(4)^*randomisation method described (yes = 1) (no = 0)*

Double blinding:

*Up to two points are given*^(2)^*: described as double blind (yes = 1) (no = 0) and*^(5)^*double blinding method described (yes = 1) (no = 0)*

Reports of withdrawals and dropouts:

*Up to one point is given*^(3)^*: Description of withdrawals (yes = 1) (no = 0)*

**Table 4:** Quality assessment of observational studies based on the Newcastle-Ottawa Scale

Study	Selection				Comparability	Outcome			Score
	A	B	C	D	E	F	G	H	
Grant 2002 [13] ^(p)^	*	*	*	*	**	*	*	*	9*
Lodise 2007 [16] ^(R)^	*	*	*	*	**	*	*	*	9*
Lorente 2009 [18] ^(R)^	*	*	*	*	**	*	*	*	9*
Rose 2011 [35] ^(R)^	*	*	*	*	**	*	*	*	9*
Yost 2011 [21] ^(R)^	*	*	*	*	*	*	*	*	8*
Pereira 2012 [23] ^(R)^	*	*	*	*	**	*	*	*	9*
Lee 2012 [24] ^(R)^	*	*	*	*	**	–	*	*	8*
Waxier 2012 [25] ^(R)^	*	*	*	*	**	–	*	*	8*
Cutro 2014 [27] ^(R)^	*	*	*	*	*	*	*	*	8*
Schmees 2016 [31] ^(R)^	*	*	*	*	**	–	*	*	8*
Winstead 2016 [30] ^(R)^	*	*	*	*	**	–	*	*	8*
Fan 2017 [34] ^(P)^	*	*	*	*	**	–	*	*	8*

^(P)^ = prospective cohort study and ^(R)^ = retrospective cohort study

Selection:

A: representation of the exposed cohort (yes = *) (no = −), B: selection of non-exposed cohort (yes = *) (no = −), C: ascertainment of exposure (yes = *) (no = −), D: demonstration that outcome of interest was not present at start of study (yes = *) (no = −)

Comparability:

E: comparability of cohorts on the basis of the design or analysis [controls for: age, sex and marital status (yes = *) (no = −) and for other factors (yes = *) (no = −)]

Outcome:

F: assessment of outcome (yes = *) (no = −), G: was follow up long enough for outcome to occur (yes = *) (no = −) and H: adequacy of follow up of cohorts (yes = *) (no = −).

### Meta-analysis of included studies

#### Clinical cure

**Fig. 2:**
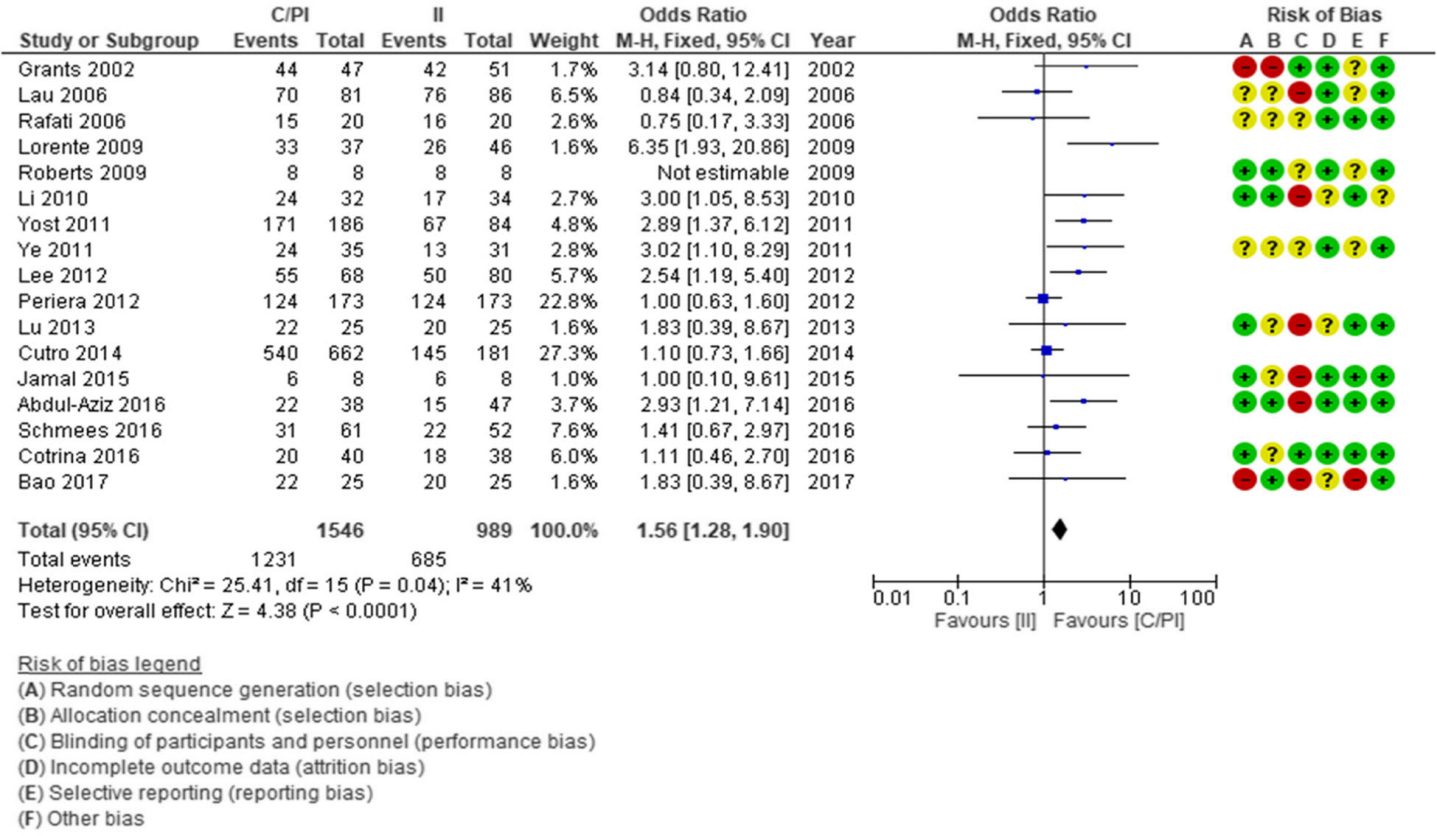
Forest plot representing the odds ratio of clinically cured patients from the C/PI and II patients in included studies

**Fig. 3:**
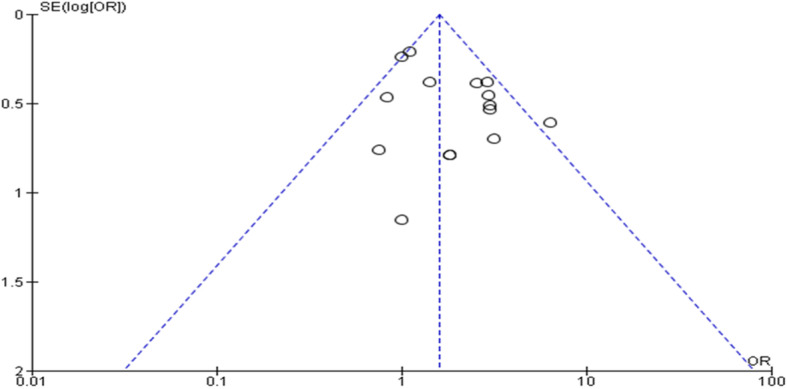
Forest plot representing the odds ratio of mortality patients from C/PI and II patients in included studies

Seventeen of the included studies reported clinical cure rates (Table 2) [6, 13–15, 18–21, 23, 24, 26–29, 31–33]. Patients that received C/PI had a statistically significantly higher clinical cure rate compared to those who received treatment via II (2535 patients; OR 1.56, 95% C.I 1.28–1.90, *P* = 0 .0001; Fig. 2). No significant heterogeneity was found among the studies (I^2^ = 41%, *P* = 0.04). The symmetrical funnel plot obtained indicates the absence of publication bias (Fig. 3).

Despite methodological differences among selected studies, patients receiving C/PI displayed higher clinical cure rates compared with patients receiving II; overall, clinical cure rate was 79.62 and 69.26% for C/PI and II respectively. Pooling results from the 17 studies that reported clinical cure showed that the odds of clinical cure was higher in patients receiving C/PI. The pooled OR shows that C/PI piperacillin-tazobactam was 1.56 (95% C.I 1.28–1.90, *P* = 0 .0001), indicating clinical cure rates are 34% higher than in II with the true population effect between 72 and 10%.

#### Mortality

**Fig. 4:**
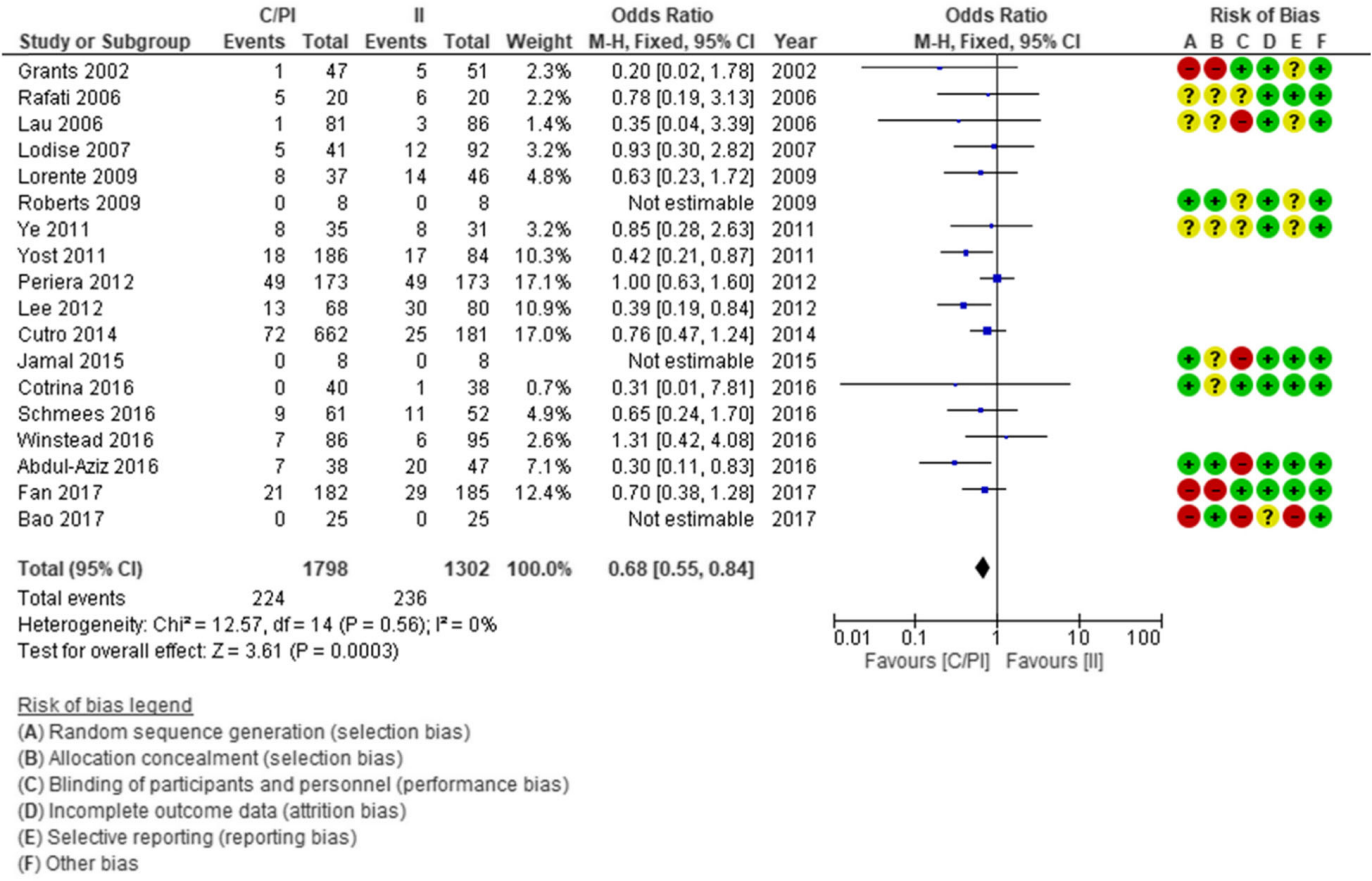
Symmetric funnel plot indicating the absence of publication bias in terms of clinical cure

**Fig. 5:**
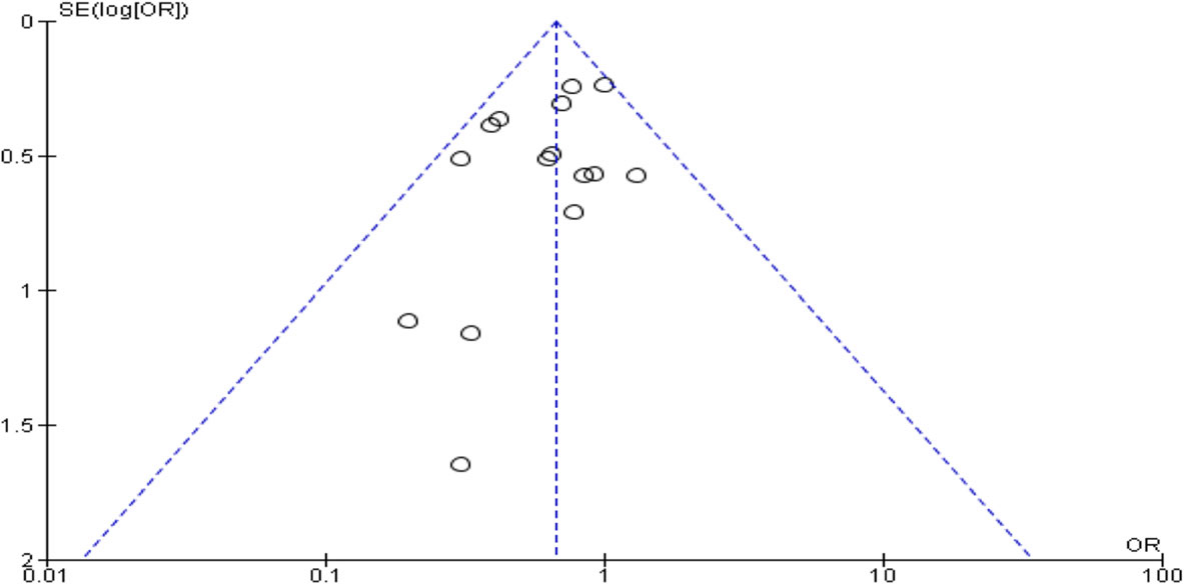
Symmetric funnel plot indicating the absence of publication bias in terms of patient mortality

Eighteen of the included studies reported patient mortality rates (Table 2) [13–18, 20, 21, 24, 27–34, 37]. Statistically significantly fewer mortality rates were found among patients receiving C/PI compared with patients receiving conventional II (3100 patients; OR 0.68, 95% C.I 0.55–0.84, *P* = 0 .0003; Fig. 4). No significant heterogeneity was found among the studies (I^2^ = 0%, *P* = 0.56). The symmetrical funnel plot obtained indicates the low possibility of publication bias (Fig. 5).

Results obtained from meta-analysis suggested that C/PI piperacillin-tazobactam resulted in significantly lower mortality rates. Overall, ICU mortality rate was 12.46 and 18.13% for C/PI and II respectively. Combining results from 18 studies that reported mortality, the pooled OR shows that C/PI piperacillin-tazobactam was 0.68 (95% C.I 0.55–0.84), indicating lower mortality rates compared with conventional II. This was statistically significant (*P* = 0.0003) with the true population effect between 84 and 55%.

#### Microbiological cure

**Fig. 6:**
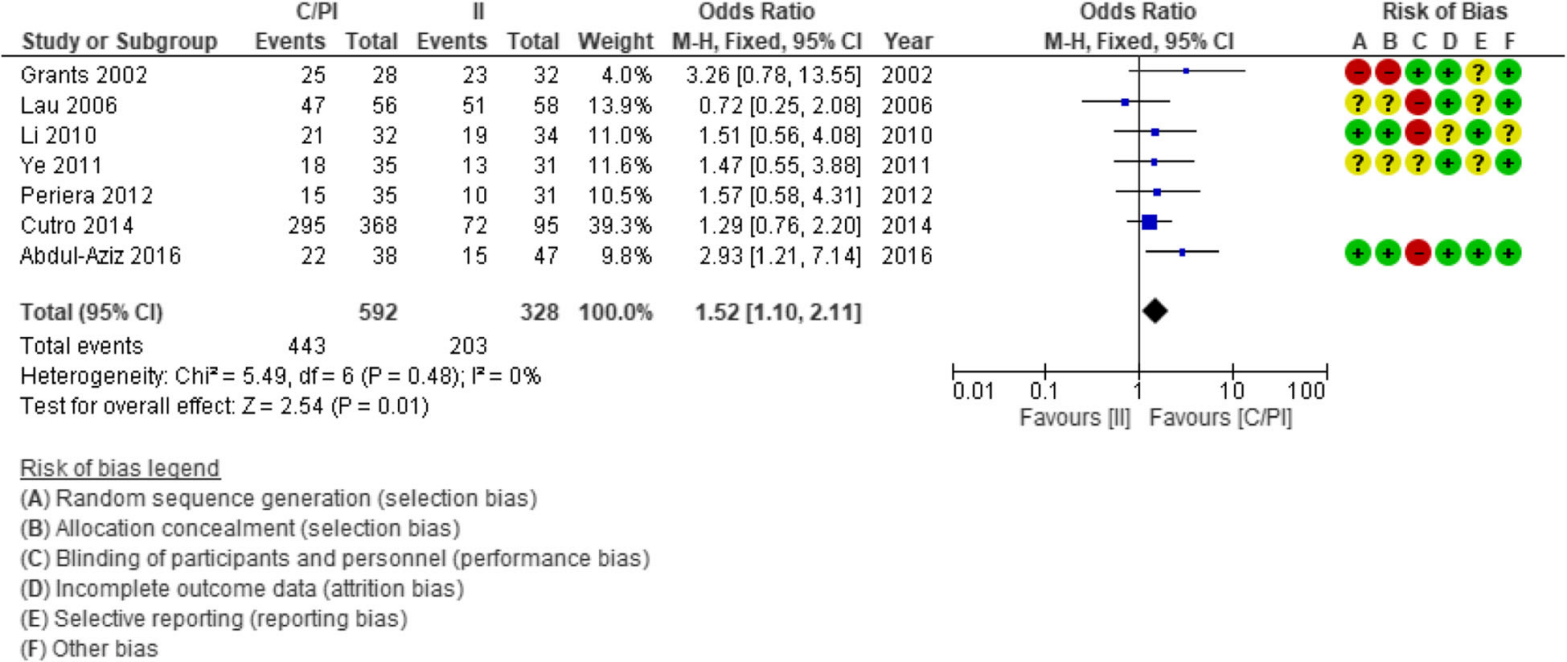
Forest plot representing the odds ratio of microbiologically cured patients from the C/PI and II patients in included studies

**Fig. 7:**
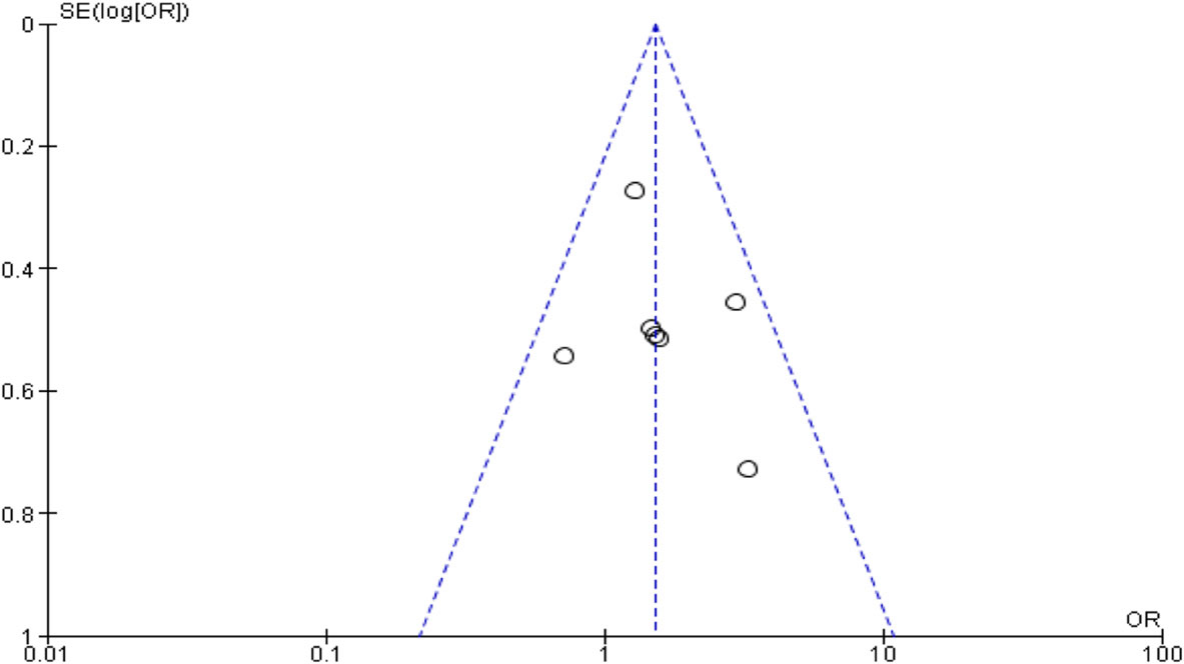
Symmetric funnel plot indicating the absence of publication bias in terms of microbiological cure

Seven of the included studies reported microbiological cure rates [13, 14, 19, 20, 23, 27, 33]. Lau et al. [14] found no statistically significant difference between the dosing regimens however, higher microbiological success was seen in patients receiving II. In contrast, Abdul-Aziz et al. [33] found C/PI piperacillin-tazobactam had significantly higher microbiological cure rates compared with II. Pooling of the outcomes of seven studies that reported microbiological cure rates showed that patients receiving C/PI had significantly higher microbiological success rates (920 patients; OR 1.52, 95% C.I 1.10–2.11, *P* = 0.01; Fig. 6). No significant heterogeneity was found among studies (I^2^ = 0%, *P* = 0.48). The symmetrical funnel plot obtained demonstrates the absence of publication bias (Fig. 7).

The pooled OR shows that C/PI piperacillin-tazobactam was 1.52 (95% C.I 1.10–2.11), indicating C/PI piperacillin-tazobactam achieved higher microbiological cure rates compared to conventional II. Overall, microbiological cure rates were 74.83 and 61.89% for C/PI and II respectively. This was statistically significant (*P* = 0.01).

#### Adverse events

Six of the included studies reported adverse events [13, 14, 31–34]. Participants enrolled in three of these studies experienced adverse event [14, 31, 32]. Lau et al’s [14], Bao et al. [32] and Schmees et al. [31] observed treatment-related adverse events in patients receiving both C/PI and II; CI: 16.9% vs II:13.6%, CI: 47.5% vs II:53.8%, CI: 76% vs II:92%, respectively. Boa [32] reported serious adverse events in 9 patients (PI:5 vs II:4), including renal failure, Tachycardia and confusion.

**Fig. 8:**
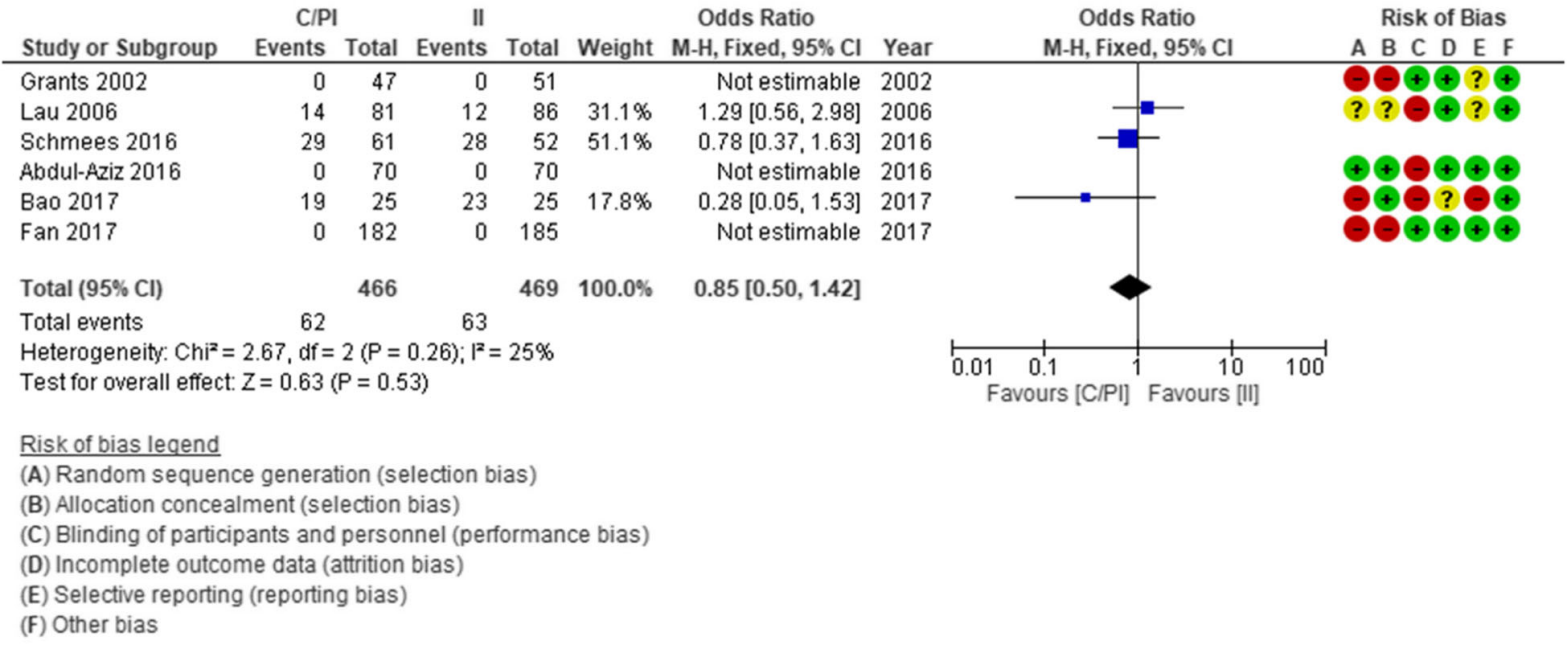
Forest plot representing the MD of length of hospital stay in C/PI and II groups in included studies

The average occurrence of adverse events was 13.3% for C/PI and 13.4% for II, respectively. Participants in the other three studies did not experience adverse events [13, 33, 34]. Data obtained from studies showed no significant difference between the two infusion strategies (935 patients; OR 0.85, 95% C.I 0.50–1.42, *P* = 0.53; Fig. 8). No significant heterogeneity was found among studies (I^2^ = 25%, *P* = 0.26).

Although adverse events were not observed in the study by Grants et al. [13], dosing and administrative errors arose where one patient was administered 13.5 g piperacillin-tazobactam dose over a 30 min II rather than a 24-h CI. Cortina et al. [29] reported that the most common side effects experienced by patients were gastrointestinal and allergic reactions but the number of patients that experienced these was not reported. The meta-analysis demonstrated that no adverse events that are directly associated to the dosing regimens occurred. C/PI resulted in a lower percentage of adverse events however, the difference between the two groups did not reach statistical significance (935 patients; OR 0.85, 95% C.I 0.50–1.42, *P* = 0.53; Fig. 8).

#### Length of hospital stay

**Fig. 9:**
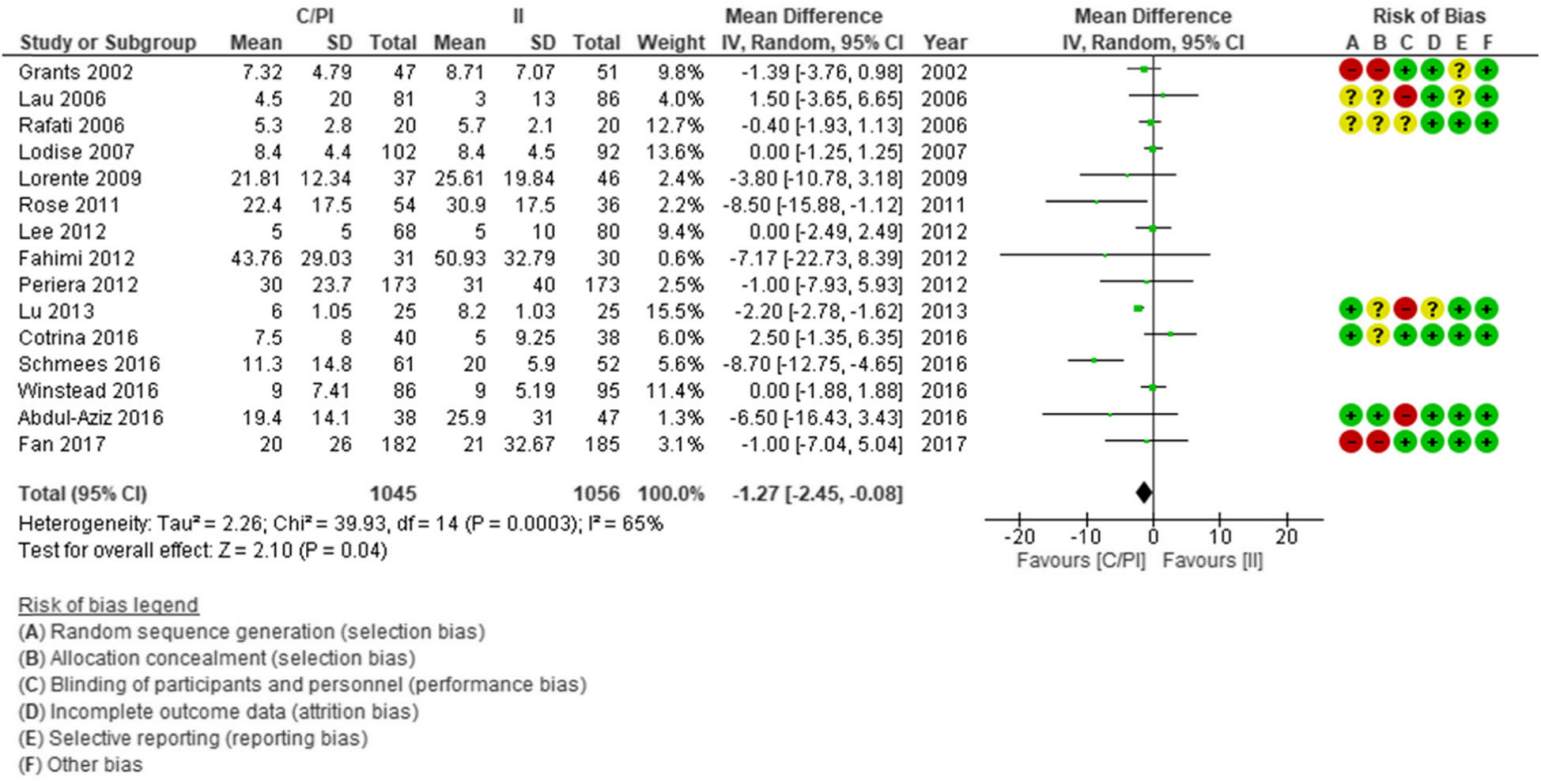
Forest plot representing the odds ratio of adverse events experienced by patients from the C/PI and II groups in included studies

Fifteen of the included studies reported length of hospital stay [13–16, 18, 23, 24, 26, 29–31, 33–35, 38]. Pooling of studies showed that patients receiving C/PI had a significantly shorter length of hospital stay (2101 patients; Mean Difference − 1.27, 95% C.I -2.45—0.08, *P* = 0.04; Fig. 9) The meta-analysis suggests there is a significant reduction in the length of hospital stay in patients receiving C/PI compared to those receiving II. Moderate heterogeneity among studies evaluating ‘length of hospital stay’ (I^2^ = 65%, *P* = 0.0003) was observed. This is likely due to clinical heterogeneity in the design and outcomes of the included studies. The length of hospital stay was an independent risk factor for mortality, however the influence of mortality on the length of hospital stay could not be evaluated.

#### Emergence of resistance

Data regarding the emergence of resistance was reported in four of the included studies [13, 14, 17, 18]. Two resistant pathogens were isolated in one study [13] however, resistant strains were not isolated in three studies [14, 17, 18] following the initiation of piperacillin-tazobactam treatment. Three studies reported that no resistant pathogen was isolated following the initiation of piperacillin-tazobactam treatment. In the study conducted by Grant et al. [13], two resistant strains were isolated from patients receiving CI piperacillin-tazobactam.

#### Risk of Bias

**Fig. 10:**
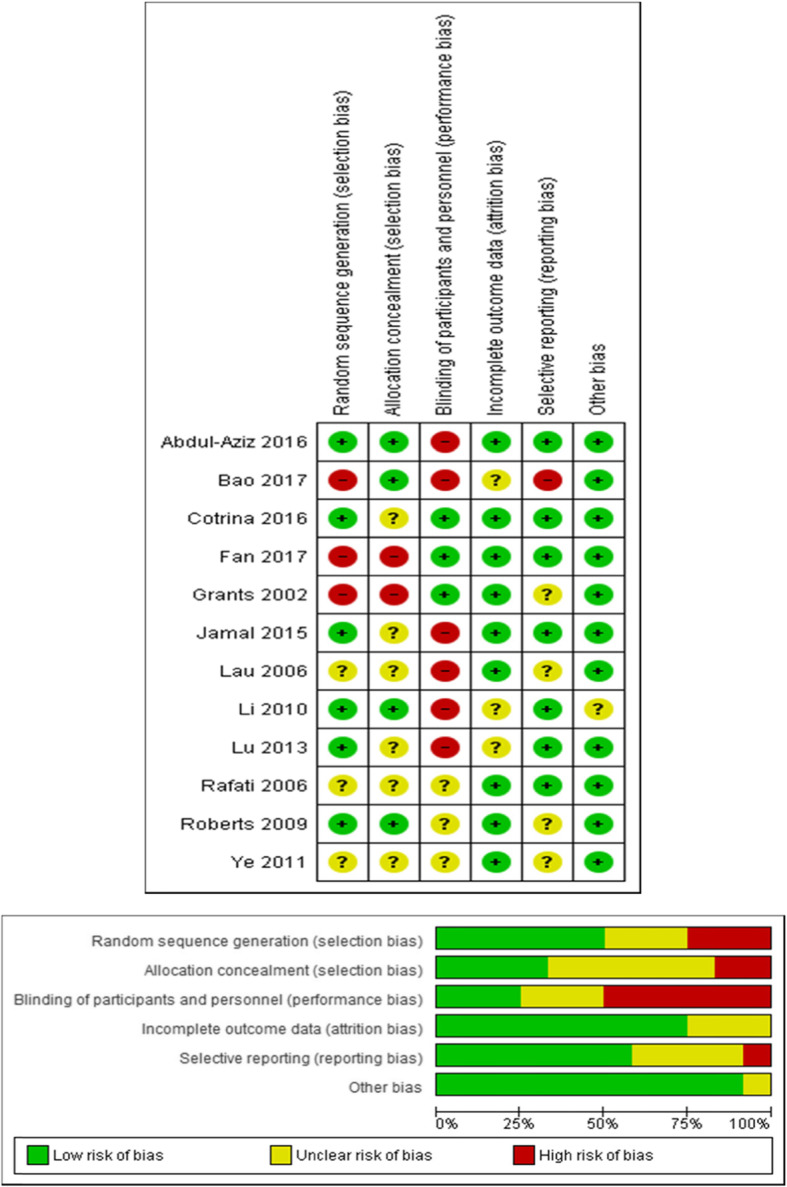
**a** Risk of bias summary of included RCT’s: displaying details about each risk of bias item for each trial. Green (+) indicates ‘low risk’, red (−) indicates ‘high risk’ and yellow (?) indicates ‘unclear risk’. **b** Risk of bias assessment displaying judgements about each risk of bias item presented as percentages across all RCT’s

The majority of RCT’s and prospective studies assessed were judged to have a low risk of bias for random sequence generation, allocation concealment, incomplete outcome data, selective reporting and other biases. However, evaluations of blinding of participants and personnel parameter was judged to have a high or unclear risk of bias (Fig. 10).

## Discussion

To the best of our knowledge, this systematic review and meta-analysis is the largest study describing clinical outcomes of severely ill patients treated with either C/PI or II piperacillin-tazobactam. The selected studies involved 3828 critically ill adult participants in total (C/PI = 2197 and II = 1631) from geographically diverse regions.

It is the first meta-analysis that shows C/PI resulted in significantly: (1) higher clinical cure rates (2) lower mortality rates (3) higher microbiological success rates and (4) decreasing the length of hospital stay specifically in critically ill patients. In all the studies, the primary outcome assessed was clinical efficacy. The current study differs from previously published systematic reviews and meta-analyses [4, 36, 39–44] as it specifically focuses on use of piperacillin-tazobactam in critically ill ICU patients. The present systematic review and meta-analysis identified a significant clinical cure, mortality, microbiological cure and length of hospital stay benefit for C/PI across all included studies.

In theory, C/PI of piperacillin-tazobactam is a broadly recognised strategy to optimize antibiotic therapy, where concentrations remain above the MIC for a higher percentage of time. Studies have demonstrated that the amount of time in which the free or non-protein bound antibiotic concentration exceeds the MIC (fT > MIC) of the organism is the best predictor of clinical and microbiologic response for β-lactams [45, 46]. However, data to backup this developing practice have been sparse [43]. Twenty-three published studies comparing C/PI and II of piperacillin-tazobactam fit the inclusion criteria (Table 2).

Outcomes of the current study correlate and expand upon previously published reviews including several analyses comparing clinical efficacy of dosing regimens for beta-lactams generally [39–42]. These studies pointed towards a more favourable outcome of C/PI for improved clinical cure and resolution of illness. Falagas et al. 2013 [40] and Vardakas el al 2018 reviewed outcomes of C/PI and II beta-lactams. There was a significant reduction in mortality rates among patients receiving C/PI in both studies. Roberts et al. 2016 [42] observed higher clinical rates and reduced mortality in C/PI patients and Lal et al. 2016 [39] found C/PI to reduce clinical failure rates.

Finding in this study are consistent with published reviews focused specifically on piperacillin-tazobactam [4, 36, 43, 44]. Yusuf el at 2014 [4] reviewed literature comparing the effectiveness of C/PI and II administration of piperacillin-tazobactam. They documented C/PI improved clinical cure, mortality and length of hospital stay in comparison to II. Yang et al. 2015/6 [36, 44] observed similar beneficial effects of C/PI in their systematic reviews. Recently, Rhodes et al. 2017 [43] evaluated a wide range of severely ill patients, from hospitalised patients to critically ill patients admitted to ICU. C/PI piperacillin-tazobactam is associated with improved clinical outcome and significantly reduced mortality rates.

Several observations were encountered from reviewing this data which led to reduced comparability among studies. First, clinical heterogeneity was present as selected studies comparing C/PI and II in terms of clinical outcomes have confounding factors including patient sample size, study settings, study design, quality, intervention and outcomes. Second, information regarding monotherapy and combination antibiotic therapy were not reported in the included studies. This reduces the validity of conclusions on C/PI, as agents used possess different antimicrobial spectrum, and drug-drug interactions were unknown hence not considered. Third, assessing safety was challenging due to under-reporting of adverse events. Higher serum concentrations in C/PI patients over a longer period could potentially result in an increased number of adverse events. Fourth, a large number of included studies were RCT’s (10/23; 43.5%) with small sample size. Small sample size may result in bias and the probability of small study effects contributing to the favourable outcome for C/PI. However, meta-analyses including small and large studies did not indicate significant discrepancies and similar outcomes were observed with fixed and random effect models. Fifth, duration of piperacillin-tazobactam administration and dosing is not homogenised between studies. CI was administered over the entire dosing interval and the duration of a PI between studies ranged between 3 and 4 h which is in line with proposed guidelines (2–4 h). Traditional II durations between studies ranged between 20 and 30 min (usually 30–60 min) [47]. Heterogeneity of dosing was also noted. In 7/23 studies piperacillin-tazobactam treatment was initiated with a loading dose to ensure rapid achievement of therapeutic concentrations. Also, the total daily dose administered differed between CI, PI and II, providing an additional confounding factor as to whether the duration of infusion or total daily dose attributed to clinical outcome (Table 2). Finally, it wasn’t apparent how critically ill the patients within studies were as only four studies reported SOFA scores.

Findings of this meta-analysis should be interpreted in view of certain limitations. First, throughout this review, PI and CI were combined and referred to as C/PI, thus, it is unclear which of the two dosing strategies is most effective for critically ill patients. Additionally, all studies were evaluated for quality and risk of bias and based on the overall assessment of these two factors no studies were excluded (Tables 3, 4 and Fig. 10). Also, a medical librarian was not involved in this study.

## Conclusion

In conclusion, C/PI of piperacillin-tazobactam in critically ill patients was associated with (1) higher clinical cure rates (2) lower mortality rates, (3) higher microbiological success rates and, (4) decreasing the length of hospital stay in critically ill ICU patients. No reduction in ‘adverse events’ and ‘emergence of resistance’ has been demonstrated. Results obtained in this study show that clinical outcome in critically ill patients is significantly better in those receiving C/PI. However, the superiority of the benefits and outcome gains achieved with C/PI administration in comparison to II is difficult to deduce as studies selected show considerable heterogeneity in terms of: (1) type of isolated bacteria, (2) piperacillin-tazobactam dose, (3) MIC of pathogen, (4) patient renal function, (5) duration of hospital stay and (6) outcome definitions. More rigorous scientific studies in critically ill patients are warranted to reach a sufficient level of evidence to promote the widespread adoption and further implementation of C/PI piperacillin-tazobactam.

## Data Availability

Data generated or analysed during this study are either included in this published article or are available from the corresponding author on reasonable request.

## Abbreviations

C.I: Confidence Interval
CI: Continuous Infusion
C/PI: Continuous/Prolonged Infusion
ICU: Intensive Care Unit
II: Intermittent Infusion
MD: Mean Difference
MIC: Minimal Inhibitory Concentration
OR: Odds Ratio
PD: Pharmacodynamics
PI: Prolonged Infusion
PK: Pharmacokinetics
RCT: Randomised Controlled Trials
